# Antiproliferative activities and phenolic acid content of water and ethanolic extracts of the powdered formula of *Houttuynia cordata* Thunb. fermented broth and *Phyllanthus emblica* Linn. fruit

**DOI:** 10.1186/s12906-018-2185-x

**Published:** 2018-04-11

**Authors:** Piyawan Kumnerdkhonkaen, Somprasong Saenglee, Md. Ali Asgar, Gulsiri Senawong, Kanoknan Khongsukwiwat, Thanaset Senawong

**Affiliations:** 1 Department of Biochemistry, Faculty of Science, Khon Kaen University, Khon Kaen, 40002 Thailand; 2 Program in Biological Science, Faculty of Science, Khon Kaen University, Khon Kaen, 40002 Thailand; 3 Natural Product Research Unit, Faculty of Science, Khon Kaen University, Khon Kaen, 40002 Thailand

**Keywords:** Anticancer activity, *Houttuynia cordata*, Phenolic acids, Powdered formula, *Phyllanthus emblica*

## Abstract

**Background:**

*Houttuynia cordata* Thunb. and *Phyllanthus emblica* Linn. are native plants with medicinal and nutritive significance in Asia. The present study was aimed at evaluating antiproliferative effects on human cancer cell lines and identifying the phenolic acid composition of water and ethanolic extracts of the powdered formula of *H. cordata* fermented broth and *P. emblica* fruit.

**Methods:**

Anticancer activity of the extracts was evaluated against HeLa, HT29, HCT116, MCF7 and Jurkat cells using an MTT assay and flow cytometric analysis of apoptosis induction and cell cycle arrest. Reverse phase HPLC was exploited for identification and quantification of some phenolic acids.

**Results:**

MTT assay showed that both water and ethanolic extracts significantly decreased the viability of cancer cells in a dose- and time-dependent fashion. Based on the IC_50_ values, ethanolic extract (IC_50_ values = 0.12–0.65 mg/mL) was more cytotoxic than water extract (IC_50_ values = 0.22–0.85 mg/mL) and Jurkat cells were the most sensitive to both extracts (IC_50_ values = 0.12–0.69 mg/mL). The underlying mechanism for antiproliferative activity was apoptosis induction, especially in HT29, HCT116, MCF7 and Jurkat cells. HT29 cells were the most sensitive to extract-induced apoptosis. Ethanolic extract was more effective at inducing apoptosis than water extract. Moreover, cell cycle arrest was found to be another mechanism behind growth inhibition in Jurkat and HCT116 cells. However, these extracts were relatively less toxic to non-cancer Vero cells. HPLC analysis demonstrated that the powder mix extracts contained seven identified phenolic acids namely gallic, *p*-hydroxybenzoic, vanillic, syringic, *p*-coumaric, ferulic and sinapinic acids, where *p*-coumaric acid was detected in the highest concentration followed by ferulic acid.

**Conclusion:**

Overall, the results of this study suggest the powdered formula of *H. cordata* fermented broth and *P. emblica* fruit as an alternative medicine for cancer prevention and treatment.

## Background

Nowadays, cancer is the leading cause of death in many countries of the world. Chemotherapy is a common treatment strategy for advanced and metastatic cancer. However, the cost of chemotherapy is a major concern in developing countries. In addition, resistance and negative side effects resulting from chemotherapy with conventional anti-cancer drugs pose some limitations in clinical use. Consequently, herbal medicine remains an inevitable alternate or adjunct therapy for cancer prevention and treatment. Various plant components with bioactive compounds and derived natural products are being intensively investigated to explore their potential for prevention and treatment of cancer. *Houttuynia cordata* Thunb. (known in Thai as Plu-Kao) is a medicinal herb indigenous to many parts of East and Southeast Asia including Thailand [1, 2]. The plant has been recognized as possessing many biological and pharmacological properties including anticancer, antioxidative, antiviral and immunomodulatory activities [3]. The fermented *H. cordata* has been demonstrated to exhibit cytotoxic effects on human leukemia cells and hepatocellular cancer cells to a considerable extent [4, 5].

*Phyllanthus emblica* Linn. or *Emblica officinalis* Gaertn. (known in Thai as Ma-kham-pom) is another medicinal plant used in Asian traditional medicine systems against different ailments including peptic ulcer, inflammation, dyspepsia and cancer, etc. [6, 7]. Its fruits are traditionally consumed for its immense nutritive values as well. Experimental evidence suggested that its fruit had several phytochemicals such as gallic acid, ellagic acid and pyrogallol that possess antineoplastic effects [6]. It has also been reported to possess immunomodulatory, chemomodulatory, radiomodulatory, antioxidative, anti-inflammatory and antimutagenic activities, which directly or indirectly prevent carcinogenesis [6, 7]. A number of in vitro and in vivo experiments point out that fruit extract of *P. emblica* has potent anticarcinogenic property [8–11]. A recent study has indicated that *P. emblica* extract inhibits ovarian cancer cell proliferation both in vitro and in vivo through inhibiting angiogenesis and inducing autophagy [12]. Furthermore, the bioactive compound gallic acid, present in fruit of *P. emblica* causes cell death through induction of apoptosis [13]. The bioactivities of *P. emblica* extract are mainly mediated by polyphenols [10]. Many phenolics have been documented to have potent antioxidant, anticancer, antibacterial, antiviral, and anti-inflammatory potentials [14]. The fruit of *P. emblica,* being rich in natural antioxidants with free radical scavenging potential, might prevent reactive oxygen species from DNA damage and carcinogenesis [10, 11, 15]. The anti-inflammatory activity of *P. emblica* extracts might deter inflammation-related cancer [10]. It has also been mentioned that ethanolic extract of *P. emblica* fruit showed antiproliferative and antioxidant activities, whereas methanol extract showed chemopreventive potential against hepatocarcinogenesis [16–18].

In recent times, the *H. cordata* fermented broth has been widely used in Thailand as a dietary supplement, however, the consumption may be limited due to its unpleasant taste. Therefore, a powdered formula of *H. cordata* fermented broth and *P. emblica* fruit has been developed. This is very interesting from a health perspective and thus, anticancer effects and chemical composition of this powdered formula needs to be investigated to provide scientific information to the public. In the present study, we evaluated antiproliferative activity of the powdered formula extracts in a panel of cancer cell lines and identified several phenolic compounds from ethanolic and water extracts of the powdered formula with HPLC and LC-MS techniques.

## Methods

### Preparation of water and ethanolic extracts

The powdered formula of *H. cordata* fermented broth and *P. emblica* fruit was obtained from the Prolac (Thailand) Co., Ltd., in Lamphun Province, Thailand. Distilled water (50 mL) or absolute ethanol (50 mL) was added to 5.0 g of the powder mix and stirred continuously for 48 h at room temperature. Subsequently, the mixture was centrifuged at 2800 x *g* and filtered through Whatman filter paper (No. 4). Finally, the water extract was lyophilized, whereas the ethanolic extract was subjected to evaporation by using rotary evaporator and blowing a stream of N_2_ gas over the sample. The water (yield = 10%; *w*/w) and ethanolic (yield = 10%; w/w) extracts were stored at − 20 °C until further use.

### Cell culture and culture condition

In this study, a human cervical cancer (HeLa) cell line was obtained from Dr. C. Pientong (Khon Kaen University, Khon Kaen, Thailand). Human breast adenocarcinoma (MCF7) and human colon cancer (HCT116) cell lines were kindly provided by Dr. O. Tetsu (University of California, San Francisco, U.S.A.). Human colon cancer (HT29) cell line was obtained from Dr. P. Picha (National Cancer Institute, Bangkok, Thailand). Human T-cell leukemia (Jurkat) and non-cancer (Vero) cell lines were kindly provided by Dr. M. Leid (Oregon State University, Oregon, U.S.A.) and Dr. S. Barusrux (Khon Kaen University, Khon Kaen, Thailand), respectively. All cells were cultured in RPMI 1640 medium supplemented with 10% fetal bovine serum, penicillin (100 U/mL), and streptomycin (100 μg/mL) (Gibco-BRL, USA) and incubated at 37 °C in a humidified atmosphere of 5% CO_2_.

### Antiproliferative activity assay

The antiproliferative effect on cancer cells was evaluated by MTT (3-(4,5-dimethylthiazol-2-yl)-2,5-diphenyltetrazolium bromide) assay. Cells (8 × 10^3^ cells/well) were seeded onto 96-well plates and incubated for 24 h to allow adherence. After 24 h, the cells were exposed to increasing concentrations (0.10–1.00 mg/mL) of water and ethanolic extracts prepared from the powder mix of *H. cordata* fermented broth and *P. emblica* fruit for 24, 48 and 72 h. Control groups were treated with double distilled water or a mixture of DMSO and ethanol (1:1). After the indicated time, medium was replaced with 110 μl of fresh medium containing MTT (0.5 mg/mL in PBS) (Sigma Chemical Co., St Louis, MO, USA) and incubated for 2 h. Formazan formed after conversion of MTT was dissolved in DMSO. The absorbance of formazan was measured with a microplate reader (Bio-Rad Laboratories, USA) at the wavelength of 550 nm with a reference wave length of 655 nm. The percentage of viable cells which corresponds to the production of formazan was calculated using the following formula:

$$ \% Cell\ viability=\left[ Sample\ \left({A}_{550}-{A}_{655}\right)/ Control\ \left({A}_{550}-{A}_{655}\right)\right]\ x\ 100 $$

### Analysis of apoptosis induction by flow cytometry

Induction of apoptosis was assayed using a FITC-Annexin V apoptosis detection kit (BioLegend, USA) in accordance with the manufacturer’s instructions. Cells (1 × 10^6^ cells) were seeded into a 4.5-cm dish and incubated for 24 h. After 24 h, cells were treated with different concentrations of the water and ethanolic extracts*.* Cells were also treated with actinomycin D (10 μg/mL) as a positive control. After 24 h, the cells were harvested, washed with PBS and centrifuged at 1750 x *g* for 2 min. The cells were resuspended in ice-cold Annexin-binding buffer, stained with Annexin V-FITC and propidium iodide (PI) solutions, and then incubated for 15 min at room temperature in the dark. Finally, stained cells were analyzed using BD FACS Calibus Flow Cytometer (Becton Dickinson, USA), serviced by the Research Instrument Center, Khon Kaen University, Thailand.

### Analysis of cell cycle arrest by flow cytometry

Cell cycle profile was determined with flow cytometry using propidium iodide (PI) staining. Cells (1 × 10^6^ cells) were seeded into a 4.5-cm dish and incubated for 24 h. After 24 h incubation, cells were treated with different concentrations of the water and ethanolic extracts for 24 h. Thereafter, cells were harvested, washed twice with cold PBS containing 0.1% glucose, and centrifuged at 1750 x *g* for 2 min. The pellet was fixed in cold ethanol at 4 °C for 1 h. The ethanol-fixed cells were centrifuged at 1750 x *g* for 2 min, washed twice with cold PBS and suspended in 250 μl PBS. To avoid double stranded RNA staining, 5 μl of RNase (F.C.-20 mg/ml) was added to the suspension and incubated at room temperature for 30 min. Finally, the cells were stained with 5 μl of PI (F.C.-50 mg/ml) for 1 h and analyzed by Flow Cytometry as described above.

### Preparation of free and esterified phenolic acids for high performance liquid chromatography (HPLC) analysis

The extraction was performed according to a previously described method with some modifications [19]. Ethanolic and water extracts prepared from 1.0 g of the powdered formula were added with 30 ml of 2 N NaOH and stirred for 12 min at room temperature. The mixture was centrifuged at 2800 x *g* for 20 min and then filtered through Whatman filter paper (No.4). The supernatant was extracted three times with 60 mL of diethyl ether with the aqueous extract containing unbound phenolics collected. The pH of the aqueous extract was adjusted to 1.5 by using 12 M HCl. The acidic aqueous solution was filtered through Whatman filter paper (No.4) and extracted three times with 60 ml of diethyl ether with the diethyl ether extract collected. The diethyl ether solution contained unbound phenolics and was dried over Na_2_SO_4_ (anhydrous) and filtered through Whatman filter paper (No.4). The filtrate was evaporated by rotary evaporation and then blowing N_2_ gas over the sample. The phenolic extract was dissolved in 1 ml of 50% methanol solution and filtered through 0.2 μm syringe filter. The phenolic acid content was analyzed by HPLC.

### Identification and quantitation of phenolic acids by HPLC

The identification of individual phenolic acids was performed using an HPLC system (Prominence LC-20A series, Shimadzu Scientific Instruments, Tokyo, Japan) and following the method of Senawong et al. [19]. The amount of each phenolic acid was analyzed using a standard curve and comparing ratios of peak areas of the phenolic acid standard and the internal standard (*m*-hydroxybenzaldehyde; 1 μg). The HPLC system consisted of Waters In-Line Degasser D6U-20A5, SIL-20AHT Autosampler, and SPD-M20A Photo Diode Array Detector. Empower software was used for data acquisition. The column used was a Waters system column C18 (4.6 mm i.d. × 250 mm, 5 μm particle diameter) coupled to a guard column. The temperature of the column was 25 °C and the mobile phase flow rate was 1.0 mL/min. The compounds were eluted by using a gradient elution of mobile phases A (100% acetonitrile) and B (1% acetic acid in deionized water) where A increased from 3% to 8% in 5 min and to 10% by 25 min and maintained at same level for 20 min, then resuming to initial conditions (3%) in 10 min maintaining this condition for 5 min before the next injection. Elutes were detected by the PDA detector at the wavelength of 280 nm. The identified phenolic acids were confirmed by liquid chromatography-mass spectrometry (LC-MS) analysis, serviced by the Center for Scientific and Technological Equipment, Suranaree University of Technology, Thailand.

### Statistical analysis

Data were expressed as mean ± standard deviation (SD) from three independent experiments with two replicates. Statistical analyses were carried out using the statistical program SPSS version 17.0 for windows (SPSS Corporation, Chicago, IL). The criterion for significant difference was set at *p* < 0.05.

## Results

### Antiproliferative activity of water and ethanolic extracts of the powdered formula of *H. cordata* fermented broth and *P. emblica* fruit

**Fig. 1:**
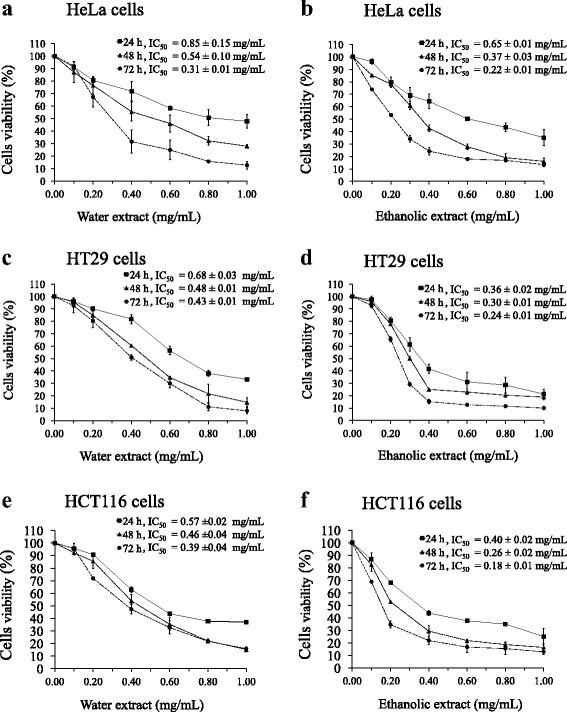
Effect of water and ethanolic extracts prepared from the powdered formula of *H. cordata* fermented broth and *P. emblica* fruit on proliferation of HeLa (**a**, **b**), HT29 (**c**, **d**) and HCT116 (**e**, **f**) cells, treated for 24, 48 and 72 h. The cell viability was calculated by comparison with the control, 0.5% DMSO treated. The results were shown as mean ± S.D. (*n* = 6). The average of half maximal inhibitory concentration (IC_50_) values calculated from 3 independent experiments was presented along with a line graph

**Fig. 2:**
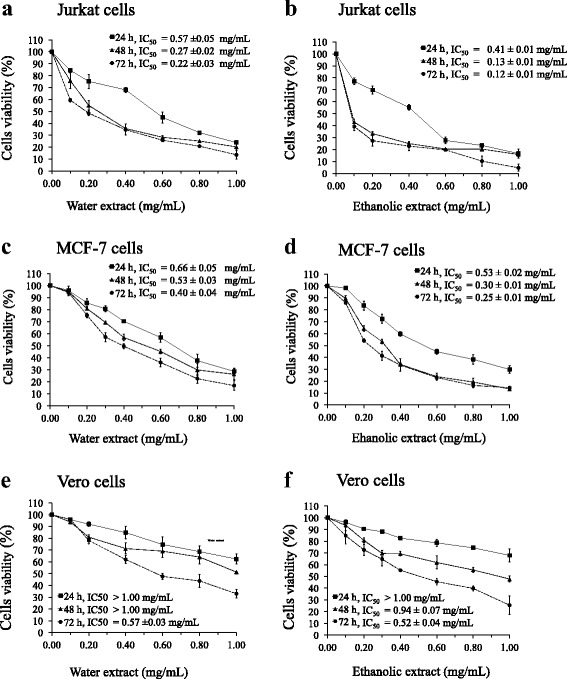
Effect of water and ethanolic extracts prepared from the powdered formula of *H. cordata* fermented broth and *P. emblica* fruit on cell proliferation of Jurkat (**a**, **b**), MCF7 (**c**, **d**) and Vero (**e**, **f**) cells, treated for 24, 48 and 72 h. The cell viability was calculated by comparison with the control, 0.5% DMSO treated. The results were shown as mean ± S.D. (*n* = 6). The average of half maximal inhibitory concentration (IC_50_) values calculated from 3 independent experiments was presented along with a line graph

Antiproliferative activity of water and ethanolic extracts of the powdered formula in a panel of cancer cell lines (HeLa, HT29, HCT116, MCF7 and Jurkat) and a non-cancer cell line (Vero) was investigated by MTT assay. From dose-response curves, it was shown that water and ethanolic extracts inhibited proliferation of all the cancer cell lines in a dose- and time-dependent manner (Figs. 1a-f and 2a-d). Overall, ethanolic extract was found to be more cytotoxic than water extract with slight variations over the period of time (Figs. 1b, d, f and 2a, d). The growth of Jurkat cells was more sensitive to both extracts in comparison to other cancer cell lines studied (Fig. 2a and b). Cellular sensitivity was determined as IC_50_ values for each cell line for each extract is presented along with the dose-response curves in Figs. 1 and 2. Cellular sensitivities differed depending on cell type and extract. The viability of the non-cancer cell line (Vero cells) was also progressively decreased with increasing concentrations of the extracts (Fig. 2a and b) but much less in comparison with the cancer cell lines.

### Induction of apoptosis by water and ethanolic extracts of the powdered formula of *H. cordata* fermented broth and *P. emblica* fruit

**Fig. 3:**
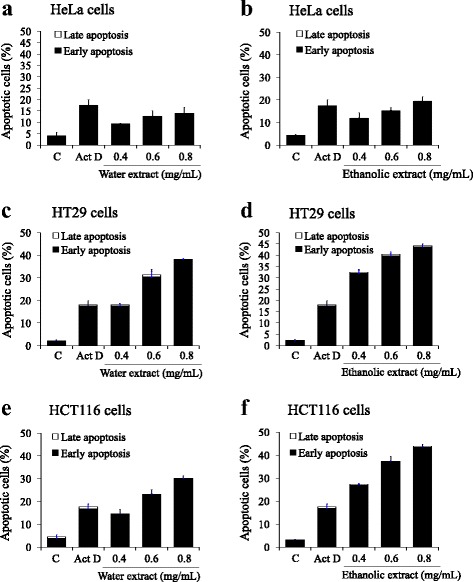
Flow cytometric analysis of apoptosis induction in HeLa (**a**, **b**), HT29 (**c**, **d**) and HCT116 (**e**, **f**) cells. Cells were treated with various concentrations of water and ethanolic extracts prepared from the powdered formula of *H. cordata* fermented broth and *P. emblica* fruit for 24 h. The Annexin V-FITC/PI staining apoptotic cells were analyzed using flow cytometry. Actinomycin D (Act D; 10 μg/mL) was used as a positive control. Bar graph shows the summarized data from three independent experiments performed in duplicate compared with untreated control (**c**)

**Fig. 4:**
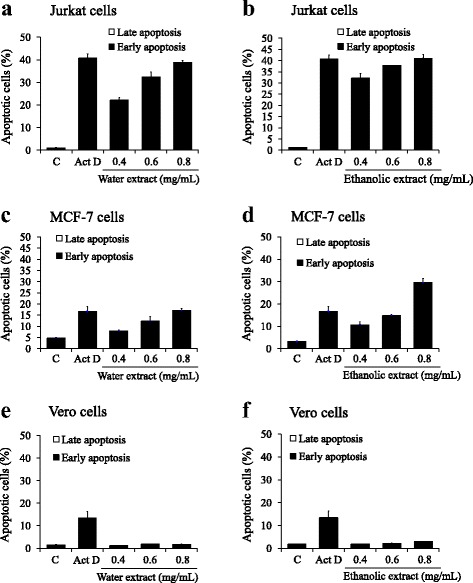
Flow cytometric analysis of apoptosis induction in Jurkat (**a**, **b**), MCF7 (**c**, **d**) and Vero (**e**, **f**) cells. Cells were treated with various concentrations of water and ethanolic extracts prepared from the powdered formula of *H. cordata* fermented broth and *P. emblica* fruit for 24 h. The Annexin V-FITC/PI staining apoptotic cells were analyzed using flow cytometry. Actinomycin D (Act D; 10 μg/mL) was used as a positive control. Bar graph shows the summarized data from three independent experiments performed in duplicate compared with untreated control (**c**)

To further investigate whether induction of apoptosis underlies the antiproliferative effect of the powder mix, apoptosis was evaluated using Annexin V-FITC staining and flow cytometry. As presented in Figs. 3 and 4, treatments with 0.40, 0.60 and 0.80 mg/mL of water and ethanolic extracts significantly increased the percentage of early apoptotic cells in HeLa, HT29, HCT116, MCF7, and Jurkat cells in a concentration-dependent manner. Ethanolic extract was found to be more effective than water extract in induction of cancer cell apoptosis. Ethanolic extract induced apoptosis was most effective in HT29 cells (43.8 ± 1.3%) followed by HCT116 (43.5 ± 1.3%) and Jurkat (40.9 ± 1.8%) cells (Figs. 3d, f and 4b, respectively). Water extract induced apoptosis was most effective in HT29 cells (38.8 ± 0.8%) followed by Jurkat (38.0 ± 0.7%) and HCT116 (29.9 ± 1.4%) cells (Figs. 3c, e, and 4a, respectively). HeLa cells were the least sensitive to the extracts among the cancer cells tested. In addition, the highest concentration (0.8 mg/mL) of all extracts showed greater cytotoxicity than actinomycin D (10 μg/mL). However, non-cancer Vero cells were found highly resistant to all extracts (Fig. 4e and f). These results indicate that apoptosis is one of the mechanisms responsible for the growth inhibition of cancer cells by the water and ethanolic extracts.

### Induction of cell cycle arrest by water and ethanolic extracts of the powdered formula of *H. cordata* fermented broth and *P. emblica* fruit

**Fig. 5:**
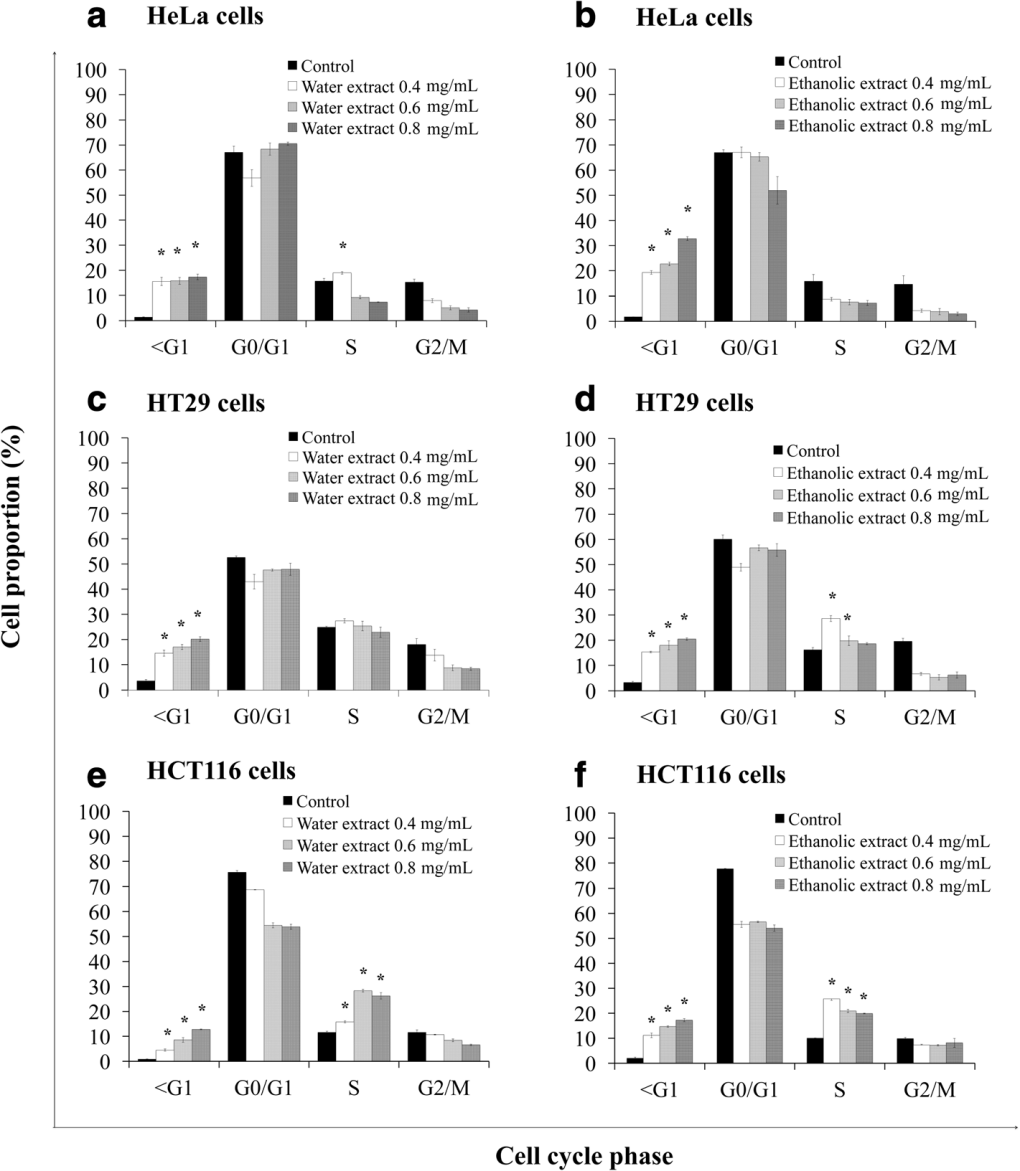
Effects of water and ethanolic extracts prepared from the powdered formula of *H. cordata* fermented broth and *P. emblica* fruit on cell cycle progression were analyzed by flow cytometry after the cells were stained with propidium iodide. For cell cycle analysis, HeLa (**a**, **b**), HT29 (**c**, **d**) and HCT116 (**e**, **f**) cells were treated with or without (control) the extracts for 24 h. Cell numbers are presented as a percentage of the total analyzed cells. Each value is presented as mean ± SD from three independent experiments

**Fig. 6:**
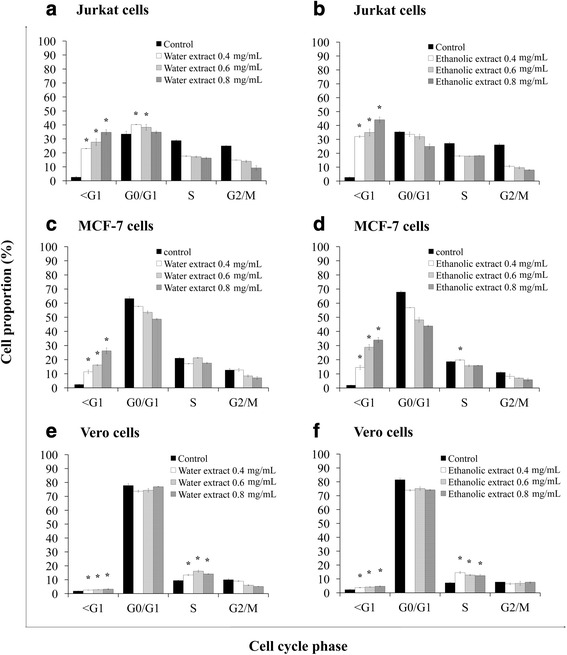
Effects of water and ethanolic extracts prepared from the powdered formula of *H. cordata* fermented broth and *P. emblica* fruit on cell cycle progression were analyzed by flow cytometry after the cells were stained with propidium iodide. For cell cycle analysis, Jurkat (**a**, **b**), MCF7 (**c**, **d**) and Vero (**e**, **f**) cells were treated with or without (control) the extracts for 24 h. Cell numbers are presented as a percentage of the total analyzed cells. Each value is presented as mean ± SD from three independent experiments

As regulation of the cell cycle is critical for the growth and progress of cancer, the effect of water and ethanolic extracts on cell cycle progression was evaluated. The results revealed that water extract induced cell cycle arrest at S phase in HeLa (Fig. 5a) and HCT116 (Fig. 5e) cells, at G0/G1 phase in Jurkat cells (Fig. 6a), and also caused a significant increase of sub-G1 fractions in these cells. However, the water extract did not affect the cell cycle progression in HT29 and MCF7 cells but only increased sub-G1 fractions when compared with solvent-treated cells (Figs. 5c and 6c, respectively). The ethanolic extract did not affect the cell cycle progression in HeLa and Jurkat cells but caused a significant increase of sub-G1 fractions (Figs. 5b and 6b, respectively). The percentages of cells at S phase in HT29 and HCT116 cells were significantly increased due to ethanolic extract-treatments (Fig. 5e and f, respectively). Ethanolic extract caused a minimal cell cycle arrest at S phase in MCF7 cells but caused a significant increase in cell death as evidenced with higher sub-G1 fraction (Fig. 6d). The water and ethanolic extracts increased the number of Vero cells in S phase and minimally increased sub-G1 population as compared with the control treatment (Fig. 6e and f).

### Identification of individual phenolic compounds by HPLC

**Fig. 7:**
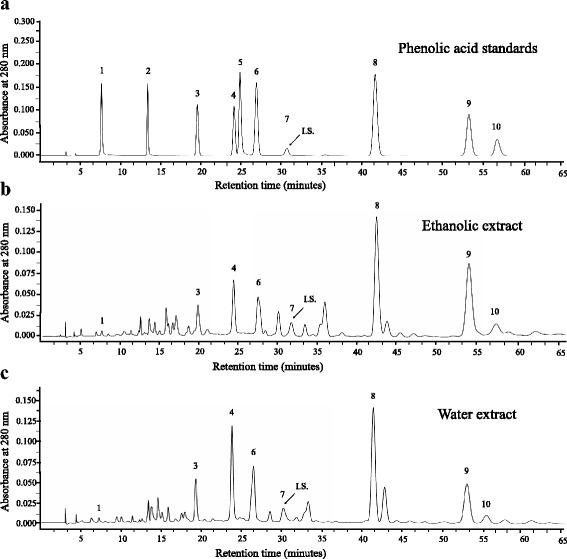
HPLC chromatograms of phenolic acid standards (**a**), base hydrolyzed ethanolic (**b**) and water (**c**) extracts of the powdered formula of *H. cordata* fermented broth and *P. emblica* fruit where 1 = gallic acid, 2 = protocatechuic acid, 3 = *p*-hydroxybenzoic acid, 4 = vanillic acid, 5 = cafeic acid, 6 = syringic acid, 7 = *p*-hydroxybenzaldehyde, 8 = *p*-coumaric acid, 9 = ferulic acid and 10 = sinapinic acid. *p*-Hydroxybenzaldehyde was used as an internal standard (I.S.)

Initial separation and identification of individual phenolic acids in the extracts of a powdered formula of *H. cordata* fermentation product and *P. emblica* fruit were carried out using a Shimadzu HPLC system. The sample chromatograms were compared with standard chromatograms (Fig. 7a) for the qualitative identification of phenolic compounds. Quantification of phenolic acids was analyzed using a standard curve and the internal standard (*m*-hydroxybenzaldehyde; 1 μg). HPLC chromatograms of phenolic acids in ethanolic and water extracts of the powder mix were demonstrated in Fig. 7b and c, respectively. Seven phenolic acids in both extracts were identified as gallic, *p*-hydroxybenzoic, vanillic, syringic, *p*-coumaric, ferulic and sinapinic acids. According to HPLC analysis, *p*-coumaric and ferulic acids were the predominant phenolic acids among the identified phenolic acids of both extracts (Table 1).

**Table 1:**
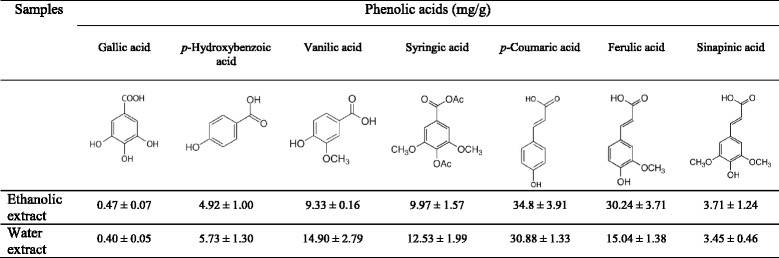
Phenolic acid compositions of base hydrolyzed extracts of the powdered formula of *H. cordata* fermented broth and *P. emblica* fruit

## Discussion

A large number of cancer patients in developing countries face problems with the high cost of chemotherapy which induces researchers to develop herbal medicine as an alternative option. Poor patients exploit traditional medicinal plants containing unknown bioactive constituents to protect their lives. Moreover, phenolic compounds present in vegetables and fruits are reported to have chemopreventive effects. Findings from previous studies suggested that the anticancer activity of the *H. cordata* fermentation products was partly attributed to the presence of *p*-coumaric, ferulic and sinapinic acids [20]. The phenolic components of *P. emblica* have been associated with inhibition of MCF7 cells in vitro [21]. Phenolic acids in *P. emblica* fruits showed strong antiproliferative activity against MK1 and HeLa cells [22]. Jose et al. reported that aqueous extract of *P. emblica* fruit showed cytotoxicity against L929 cells in a dose-dependent manner and reduced tumor volume of the solid tumors [9]. They also reported that antitumor activity of the extract may be partially related to its ability to regulate cell cycle progression. Accordingly, one may envision the powdered formula of *H. cordata* fermented broth and *P. emblica* fruit as a combination formula with promising anticancer activity. To our knowledge, we are the first who demonstrated that the powdered formula of *H. cordata* fermented broth and *P. emblica* fruit had effects on cell growth, apoptosis and cell cycle arrest. Most interestingly, both ethanolic and water extracts of the powdered formula were more potent against all cancer cell lines tested than the lyophilized water-soluble constituents of *H. cordata* fermented broth alone [20]. This observation may be related to the greater amounts of phenolic compounds especially *p*-coumaric and ferulic acids in the extracts (Table 1) compared with that in the lyophilized water-soluble constituents of *H. cordata* fermented broth alone [20].

Our results indicated that the water and ethanolic extracts reduced viability of HeLa, HT29 and MCF7 cells mainly by triggering apoptosis. Though all the cancer cells underwent apoptosis due to treatment with the extracts, antiproliferative effects of HCT116 and Jurkat cells were linked with cell cycle arrest at S and G0/G1 phases, respectively. In line with our findings, lyophilized water-soluble constituents of fermentation products from *H. cordata* suppressed the growth of HeLa, HCT116, and HT29 cells in a concentration- and time-dependent manner by inducing apoptosis [20]. Previous studies showed that *P. emblica* could induce apoptosis in mouse and human carcinoma cell lines including Dalton’s lymphoma ascites and CeHa cell lines, HeLa cells, SK-OV3 and HepG2, A549, SW620 [11, 23]. Growth inhibitory activity of *P. emblica* fruit extract was primarily demonstrated through induction of apoptotic cell death. *P. emblica* fruit extract induced apoptosis in HeLa cells by a death receptor-mediated mechanism and invasiveness of MDA-MB-231 cells in vitro*.* In addition, application of *P. emblica* extract on mouse skin resulted in reduction of tumor numbers and volumes [11]. Its anticancer activity was also related with inhibition of activator protein-1 (AP-1) which targeted transcription of viral oncogenes responsible for cervical cancer [24]. It has been reported that polyphenols enriched *P. emblica* berry extract or simple aqueous extracts have shown cytotoxic activity against ovarian cancer cells [25]. Zhu et al. mentioned that the extract of *P. emblica* containing polyphenols was capable of inhibiting cell proliferation in HeLa cells, accompanied by cell cycle arrest at G2/M phase and apoptotic cell death [26].

Moreover, in the present study, several phenolic acids were identified in the powdered formula extracts and their concentration was determined. The identified phenolic acids of both water and ethanolic extracts of the powdered formula were gallic, *p*-hydroxybenzoic, vanillic, syringic, *p*-coumaric, ferulic and sinapinic acids where the amount of *p*-coumaric and ferulic acids were greater than other identified compounds (Table 1). Similar to our study, a number of phenolic acids including protocatechuic, *p*-hydroxybenzoic, and vanillic, syringic, *p*-coumaric, ferulic, and sinapinic acids were identified in whole plant methanolic extract and water-soluble constituents of *H. cordata* fermentation products [20, 27]. Water extract of *P. emblica* was found to contain polyphenol contents [11, 28]. Many studies showed that phenolic acids were able to inhibit cancer cell growth by induction of apoptosis and/or cell cycle arrest. Gallic acid has been reported to inhibit the growth of prostate cancer cell lines (IC_50_ values = 15.6–20.7 μg/mL for 48 h exposure) [29]. *p*-Hydroxybenzoic acid has not yet been shown to suppress the growth of any cancer cell line, however, its derivatives exhibited anticancer activity [30]. *p*-Coumaric acid inhibited the growth of the colon cancer cell lines (HCT-15 and HT29 cells; IC_50_ values = 1400 and 1600 μM, respectively, for 48 h exposure) by triggering apoptosis in an ROS-dependent mitochondrial pathway [31]. Vanillic acid has not yet been shown to suppress the growth of any cancer cell line, however, vanillin has been shown to induce both apoptosis and cell cycle arrest in HT29 cells [32]. Syringic acid has been found to have anticancer potential against T47D breast cancer cells [33]. Sinapinic acid inhibited the growth of several human cancer cells (HT29, HCT116, HeLa and Jurkat cells; IC_50_ values = 1.6, 2.1, 0.9 and 2.8 mM, respectively, for 72 h exposure) [19]. Ferulic acid has been shown to inhibit the growth of several human cancer cells (HT29, HCT116, MCF7 and HeLa cells; IC_50_ values = 2.2, 2.1, 4.0 and 0.9 mM, respectively, for 72 h exposure) by apoptosis induction [34, 35]. Accordingly, gallic, vanillic, syringic, *p*-coumaric, ferulic and sinapinic acids may, at least in part, contribute to antiproliferative activity of the extracts from the powder mix of *H. cordata* fermented broth and *P. emblica* fruit especially those with relatively high amounts in the extracts (*p*-coumaric and ferulic acids). Our findings on Vero cells were in agreement with the report by Ngamkitidechakul et al. who demonstrated *P. emblica* extracts were non-toxic against normal lung fibroblast MRC5 cells [11]. Further investigation on the use of these extracts in combination with other chemotherapeutics and natural products may help to develop more effective therapeutic approaches.

## Conclusion

The present study demonstrated that ethanolic and water extracts from the powdered formula of *H. cordata* fermented broth and *P. emblica* fruit were able to prevent cancer cell growth through apoptosis and cell cycle arrest. This powdered formula is a good source of phenolic compounds especially *p*-coumaric and ferulic acids. Phenolic acids were perhaps responsible for growth inhibition of cancer cells. From the findings in this study, further research should be conducted on the use of powdered formula of *H. cordata* fermented broth and *P. emblica* fruit as a complementary medicine for cancer treatment and prevention in both animal models and clinical trials.

## Abbreviations

DMSO: Dimethylsulfoxide
HPLC: High performance liquid chromatography
MTT: 3-(4,5-dimethylthiazol-2-yl)-2,5-diphenyltetrazolium bromide
PBS: Phosphate Buffered Saline
